# Ceria nanoparticles ameliorate renal fibrosis by modulating the balance between oxidative phosphorylation and aerobic glycolysis

**DOI:** 10.1186/s12951-021-01122-w

**Published:** 2022-01-04

**Authors:** Mengling Wang, Feng Zeng, Fengling Ning, Yinhang Wang, Shilin Zhou, Jiaqi He, Cong Li, Cong Wang, Xiaolin Sun, Dongliang Zhang, Jisheng Xiao, Ping Hu, Svetlana Reilly, Hong Xin, Xudong Xu, Xuemei Zhang

**Affiliations:** 1 Department of Pharmacology, School of Pharmacy, Minhang Hospital, Fudan University, Shanghai, 201203 China; 2 Artemisinin Research Center, Institute of Science and Technology, The First Clinical Medical School, Lingnan Medical Research Center, The First Affiliated Hospital, Guangzhou University of Chinese Medicine, Guangzhou, 510405 China; 3 Key Laboratory of Smart Drug Delivery, Ministry of Education, School of Pharmacy, Fudan University, Shanghai, China, Academy for Engineering and Technology, Fudan University, 20 Handan Road, Yangpu District, Shanghai, 200433 China; 4 Science and Technology Innovation Center, Guangzhou University of Chinese Medicine, Guangzhou, 510405 China; 5 Division of Cardiovascular Medicine, Department of Medicine, University of Oxford, John Radcliffe Hospital, RadcliffeOxford, UK

**Keywords:** Ceria nanoparticles, Metabolic reprogramming, Oxidative phosphorylation, Aerobic glycolysis, Renal fibrosis

## Abstract

**Background and aims:**

Renal fibrosis is the common outcome in all progressive forms of chronic kidney disease. Unfortunately, the pathogenesis of renal fibrosis remains largely unexplored, among which metabolic reprogramming plays an extremely crucial role in the evolution of renal fibrosis. Ceria nanoparticles (CeNP-PEG) with strong ROS scavenging and anti-inflammatory activities have been applied for mitochondrial oxidative stress and inflammatory diseases. The present study aims to determine whether CeNP-PEG has therapeutic value for renal fibrosis.

**Methods:**

The unilateral ureteral obstructive fibrosis model was used to assess the therapeutic effects in vivo. Transforming growth factor beta1-induced epithelial-to-mesenchymal transition in HK-2 cells was used as the in vitro cell model. The seahorse bioscience X96 extracellular flux analyzer was used to measure the oxygen consumption rate and extracellular acidification rate.

**Results:**

In the present study, CeNP-PEG treatment significantly ameliorated renal fibrosis by increased E-cadherin protein expression, and decreased α-SMA, Vimentin and Fibronectin expression both in vitro and in vivo. Additionally, CeNP-PEG significantly reduced the ROS formation and improved the levels of mitochondrial ATP. The seahorse analyzer assay demonstrated that the extracellular acidification rate markedly decreased, whereas the oxygen consumption rate markedly increased, in the presence of CeNP-PEG. Furthermore, the mitochondrial membrane potential markedly enhanced, hexokinase 1 and hexokinase 2 expression significantly decreased after treatment with CeNP-PEG.

**Conclusions:**

CeNP-PEG can block the dysregulated metabolic status and exert protective function on renal fibrosis. This may provide another therapeutic option for renal fibrosis.

**Graphical Abstract:**

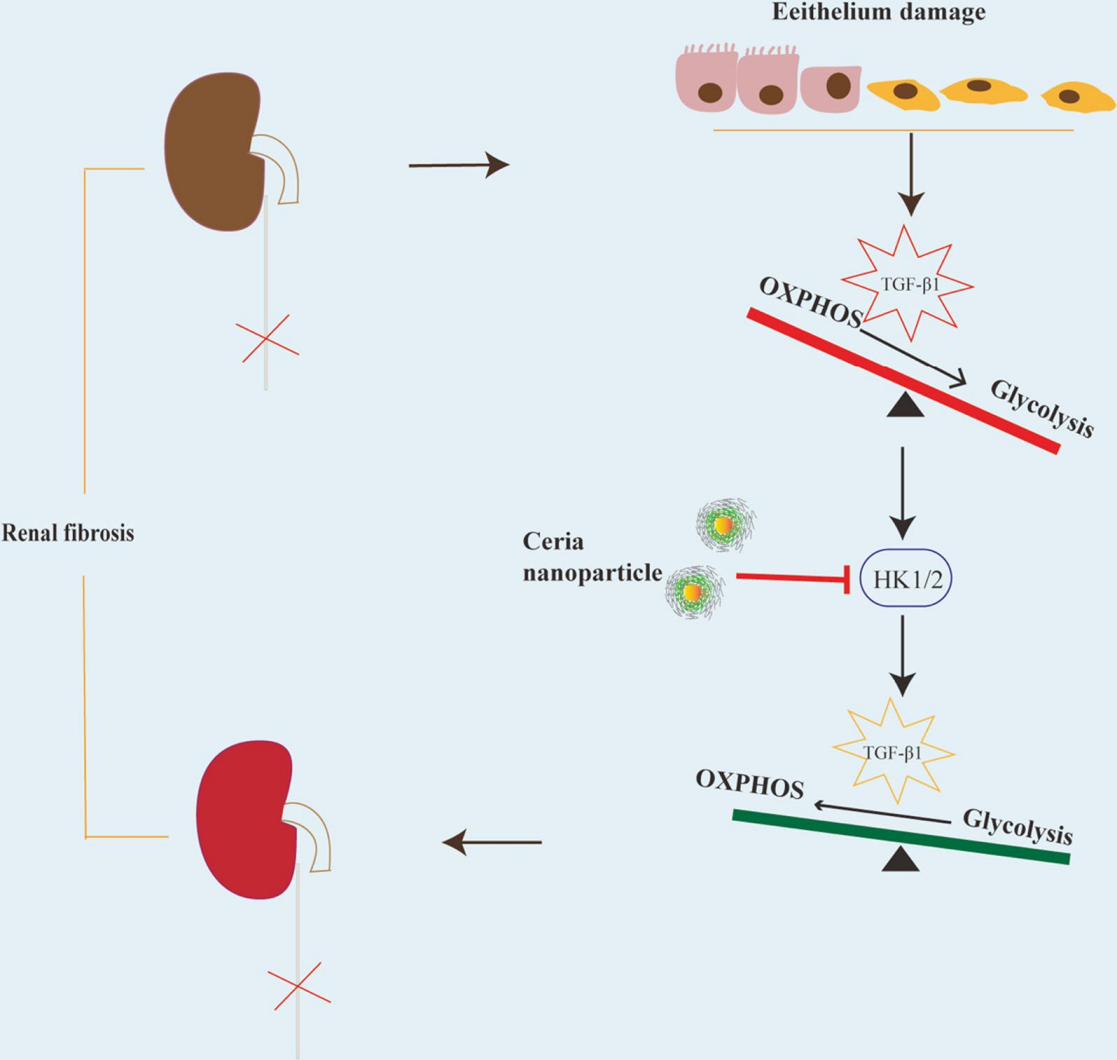

**Supplementary Information:**

The online version contains supplementary material available at 10.1186/s12951-021-01122-w.

## Background

Chronic kidney disease (CKD) is a serious common health problem worldwide, with a high incidence and mortality [1]. Renal fibrosis is the common outcome in all progressive forms of CKD. Renal fibrosis is a complicated clinical condition, which is characterized as loss of capillary networks, the accumulation of extracellular collagens, and the activation of myofibroblasts [2]. Unfortunately, the mechanism of renal fibrosis remains unclear. As a consequence, there is an urgent need to provide new opportunities for developing treatments for renal fibrosis.

The complicated pathogenesis of renal fibrosis has been studied for a long time, but has remained largely unexplored. During the development of renal fibrosis, the epithelial-to-mesenchymal transition (EMT) in tubular cells was detected [3], and this has been considered as the reactive process that occurs in response to nutrient deprivation, tissue damage, and the increase in pro-inflammatory cytokines or reactive oxygen species (ROS) [4, 5]. Furthermore, recent studies have indicated the increasing prevalence of the EMT process was associated with dysfunctional metabolic pathways through switching its metabolism manner from oxidative phosphorylation (OXPHOS) to glycolytic metabolism [6, 7]. Glycolysis is stimulated by hypoxia, and is also upregulated in the presence of oxygen as an abnormal form—the aerobic glycolysis (Warburg effect). This is a multiple-step metabolic process in response to glucose uptake, increased glycolysis and weakened oxidative phosphorylation, and its enzymatically catalyzed steps convert glucose to pyruvate molecules for cellular energy generation [8]. Hexokinase 1 (HK1) and Hexokinase 2 (HK2) are HK isoenzymes that participate in the proximal step of glycolysis, and these have been recently reported to have a significant role in glycolysis, during the development of renal fibrosis [9, 10]. Furthermore, glycolytic changes cause mitochondrial dysfunction, manifesting the influence of oxidative stress [11]. ROS is released as by-products in aerobic metabolism, which is usually associated with oxidative metabolism as well as increasingly appreciated functions in the pathophysiology of renal fibrosis [12]. Consequently, small molecular antioxidants or nanoparticles that can reduce oxidative stress may exhibit positive effects on renal fibrosis.

Existing nanosystems with different physicochemical properties for renal fibrosis treatments have been recently intensively reviewed. More than 10 nanoplatforms may be applied for targeted therapeutic delivery to the kidney, including liposomes, polymeric micelles, quantum dots, gold nanoparticles etc. [13]. Recent developments in nanomedicines have given rise to several promising compounds that exhibit positive effects on scavenge ROS to prevent renal injury [14]. In the past decades, a number of studies have demonstrated that ceria nanoparticles (CeNP) exhibit strong ROS scavenging activity and anti-inflammatory activity [15]. Consequently, CeNP may be useful in the prevention and/or treatment of liver disease [16], cardiomyopathy [17], acute kidney injury [18], stroke and neurodegenerative disorders [19]. In our previous studies have shown that CeNP-PEG can scavenge ROS triggered by stress stimuli in microglial BV-2 cell [20]. These findings increase the possibility of utilizing pro-resolving substances as a novel therapeutic strategy.

In the present study, Polyethylene glycol (PEG)-capped CeNP (CeNP-PEG) were developed. However, it remains unknown whether CeNP-PEG has therapeutic value in renal fibrosis. It was hypothesized that CeNP-PEG may exert its anti-fibrotic effect by blocking the dysregulated metabolic status of renal fibrosis—a metabolic alteration correlated with the development of the EMT progress and kidney fibrosis through its antioxidant activity. The investigators attempted to evaluate the influence of CeNP-PEG in vivo and in vitro respectively. For in vivo studies, a classical model of Unilateral ureter obstruction (UUO)-induced renal tubulointerstitial fibrosis was chosen to simulate the clinical symptoms of patients. Transforming growth factor beta (TGF-β1) is a key mediator for EMT, which stimulates renal fibrosis and inflammation. At present, researches have demonstrated that TGF-β1 induces a metabolic reprogramming in renal epithelial cells, specifically in weakened OXPHOS and increased glycolysis [10]. At the cellular level, TGF-β1-induced EMT in HK-2 cells aimed at enhanced glycolysis was initially utilized, in order to explore the relationship between CeNP-PEG, metabolic reprogramming and renal fibrosis.

## Results

### Synthesis and characterization of CeNP and CeNP-PEG

**Fig. 1:**
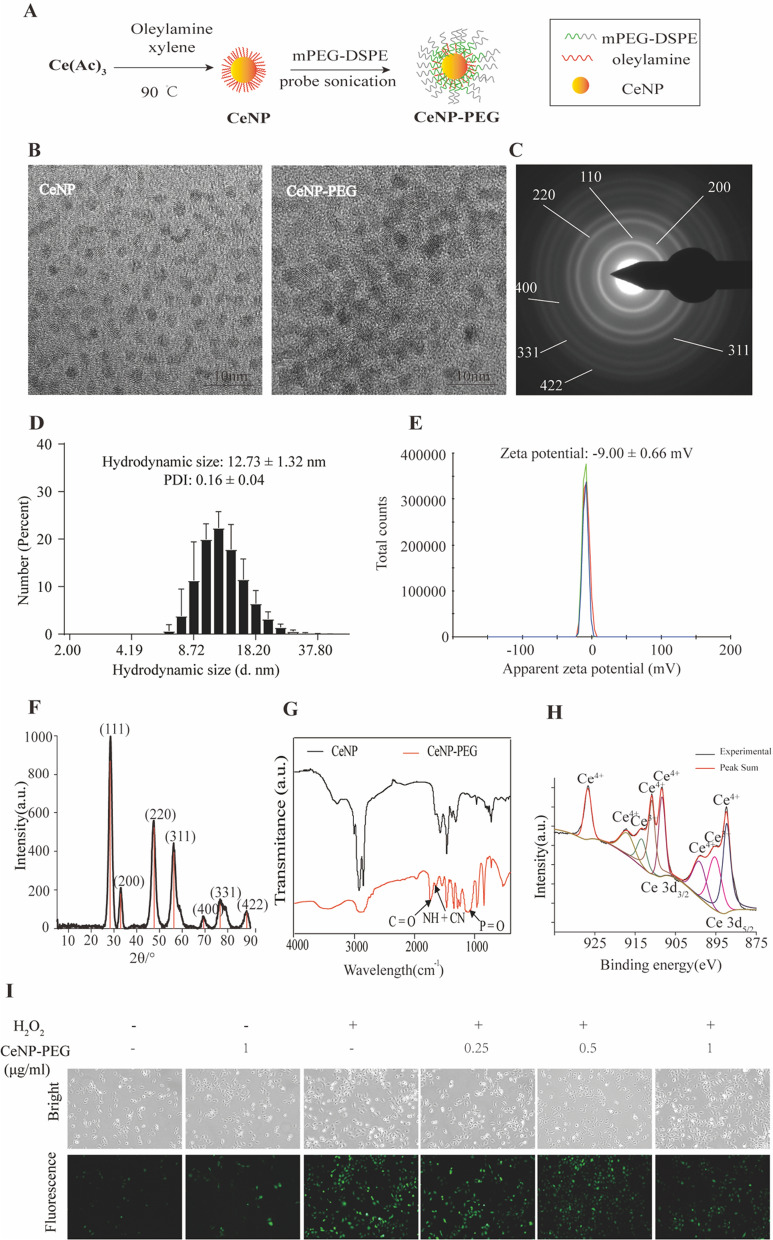
Synthesis and characterization of CeNP and CeNP-PEG. **A** Synthetic procedure of CeNP and CeNP-PEG. **B** Representative high resolution TEM images of CeNP and CeNP-PEG. The SAED **C** and XRD **F** images reveal the cubic fluorite structure of the CeNP. The hydrodynamic size **D** and zeta potential **E** of CeNP-PEG. **G** Fourier transform infrared (FT-IR) spectra of CeNP and CeNP-PEG. **H** The XPS spectra of ceria nanoparticles reveal the valence state and the corresponding binding energy peaks of 885.2 and 903.4 eV for Ce^3+^ and 882.3, 889.0, 898.4, 900.9, 907.0 and 916.8 eV for Ce^4+^. a.u.: arbitrary units. **I** Confocal images of extracellular ROS levels in the presence of CeNP-PEG treatments in HK-2 cells

The procedure for the synthesis of metallic nanozyme CeNP and CeNP-PEG are illustrated in Fig. 1A. The ultrasmall CeNP were fabricated using the reverse micelle method, followed by coating with DSPE-PEG^2k18^. As shown in Fig. 1B, the representative TEM image revealed that these CeNP were spherical, had highly crystalline and cross-lattice patterns, and had a core diameter of 2.7 nm. The size and morphology of the CeNP-PEG was similar to that of the CeNP (Fig. 1C). However, due to the PEG coating on the surface of the CeNP, the crystal lattice vanished. Attributed to the coated PEG molecules and water shells of the CeNP-PEG in an aqueous environment, the hydrodynamic size was higher than the TEM size (Fig. 1D). The hydrodynamic diameters of CeNP-PEG within 10 days were measured using DLS to evaluate their colloidal stability. As shown in supplementary Fig. 1, the hydrodynamic diameters of CeNP-PEG did not significant change, indicated that they did not obvious agglomeration or degradation within 10 days in the physiological medium and exhibited excellent colloidal stability. The XRD spectra indicated the well-indexed diffraction peaks of (111), (200), (220), (311), (400), (331) and (422) for CeNP, which were in agreement with the standard cubic fluorite structure characteristics of Ce (JCPDS card No. 34–0394) (Fig. 1F). Compared with CeNP, the absorption band in the FTIR spectrum of the CeNP-PEG sample was approximately 1738.P42 cm^−1^, and 1645.95 cm^−1^, and 1111.28 cm^−1^, and these correspond to the ν_C=O_, ν_NH_ + ν_CN_, and ν_P=O_ stretching vibration, respectively, demonstrating that the DSPE-PEG have been successfully coated on the surface of the CeNP (Fig. 1G). The SAED pattern provides further evidenced for highly crystalline CeNP (Fig. 1C). The XPS revealed the mixed-valence state of Ce^3+^ (peaks at 885.2 and 903.4 eV) and Ce^4+^ (peaks at 882.3, 889.0, 898.4, 900.9, 907.0 and 916.8 eV) (Fig. 1H). The ultrasmall CeNP exhibited excellent multienzyme-like activities, and pronounced ROS-scavenging activity [20]. Based on these results, the ROS-scavenging ability of CeNP-PEG in vitro was determined by fluorescence spectrophotometry. As shown in Fig. 1I, higher ROS levels were detected in HK-2 cells after treatment with H_2_O_2_. However, when the cells were treated with H_2_O_2,_ and received 0.5 μg/mL and 1 μg/mL of CeNP-PEG, the induction of ROS formation was significantly inhibited.

### CeNP-PEG ameliorated the phenotypic transition and fibrosis after UUO in vivo

**Fig. 2:**
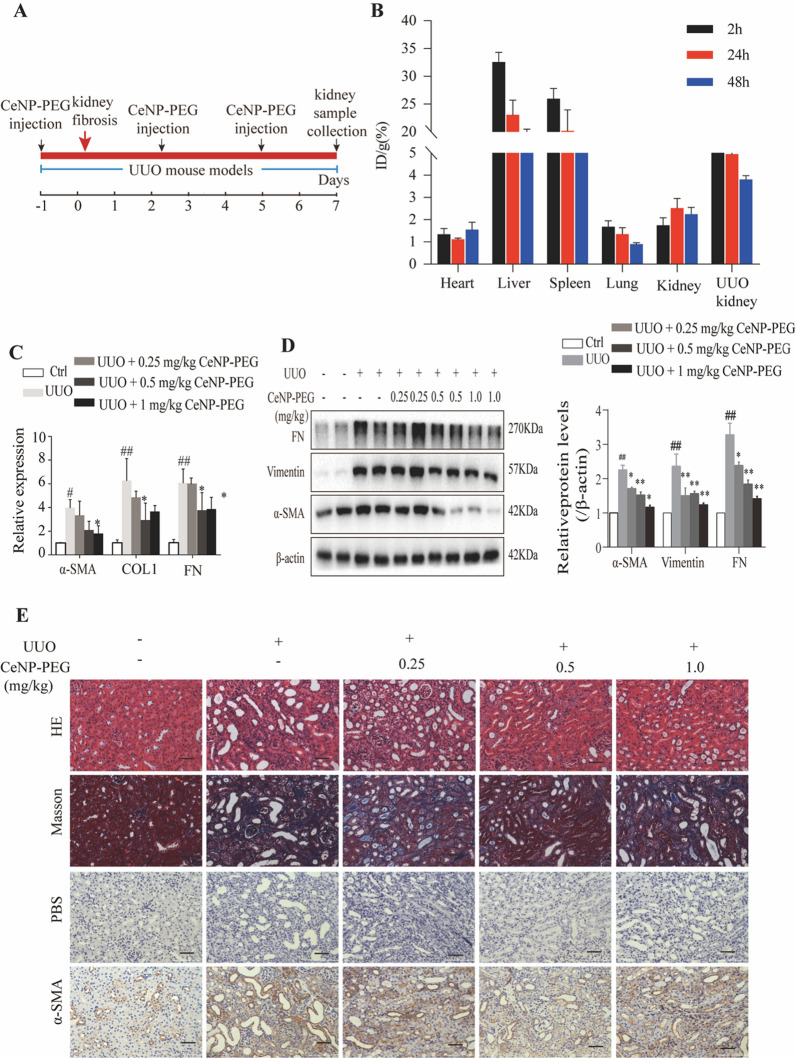
CeNP-PEG ameliorated renal fibrosis in UUO mice. **A** Schematic graph of the experimental design. **B** The biodistribution of CeNP-PEG in tissues of UUO mice. **C** The expression of α-SMA, COL1 and FN was evaluated by RT-PCR analysis. β-actin was used as the control (^#^*P* < 0.05 and ^##^*P* < 0.01 for UUO *vs.* control, and *P < 0.05 and **P < 0.01 for UUO *vs.* UUO + CeNP-PEG; *n* = 3 for each group). **D** The expression of α-SMA, Vimentin and FN was evaluated by western blot analysis. β-actin was used as the loading control (^#^*P* < 0.05 and ^##^*P* < 0.01 for UUO *vs.* control, and *P < 0.05 and **P < 0.01 for UUO *vs.* UUO + CeNP-PEG; *n* = 3 for each group). **E** The renal injury was evaluated by H&E staining. The renal fibrosis was evaluated by Masson trichrome and immunohistochemical analysis for the α-SMA expression. PBS was used as the negative control

In the present study, the investigators determined the protective role of CeNP-PEG in the renal fibrosis mice model, that is, the UUO mice model. Initially, we evaluated the systemic toxicity of CeNP-PEG. The result of the H&E sections of major organs demonstrated no significant toxicity after CeNP-PEG treatment (Supplementary Fig. 2). The biodistribution of CeNP-PEG in the major organs of UUO mice were investigated. The present study revealed that CeNP-PEG rapidly accumulate in the UUO kidney tissues and contralateral kidney tissues within 2 and 24 h after intravenous injection (Fig. 2B). Compared to contralateral kidney, CeNP-PEG in the UUO kidney were significantly increased, and remained for longer period of time, which was beneficial for kidney fibrosis treatment. Consistent with previous studies [21], the present results also revealed that CeNP-PEG accumulated in the liver and spleen, and are barely detected in heart and lung tissues of the UUO mice at different time points (Fig. 2B). Next, the investigators evaluated the effect of CeNP-PEG on different concentrations (0, 0.25, 0.50 and 1.00 mg/kg) on UUO mice, and compared this with that for untreated UUO mice and sham mice. The UUO operation resulted in the considerable increased in myofibroblast marker α-SMA and mesenchymal cell markers Vimentin and Fibronectin (FN) mRNA (Fig. 2C) and protein (Fig. 2D) expression. For UUO animals that received 0.5 mg/kg and 1 mg/kg of CeNP-PEG, the α-SMA, Vimentin and FN mRNA (Fig. 2C) and protein (Fig. 2D) levels significantly decreased, when compared to those in untreated UUO mice. In order to measure the collagen deposition in the tubulointerstitial, the kidneys were stained with Masson staining (Fig. 2E). UUO mice presented with a significantly elevated Masson staining, while the staining dramatically decreased in kidneys obtained from UUO mice that were treated with 0.5 mg/kg and 1 mg/kg of CeNP-PEG. As shown in Fig. 2E, the elevated α-SMA levels in fibrotic kidneys were further confirmed by immunohistochemical staining, and the α-SMA expression significantly decreased after treatment with CeNP-PEG. These suggest that CeNP-PEG ameliorated the kidney fibrosis after UUO in vivo.

### CeNP-PEG ameliorated the profibrogenic phenotypes induced by TGF-β1 in HK-2 cells

**Fig. 3:**
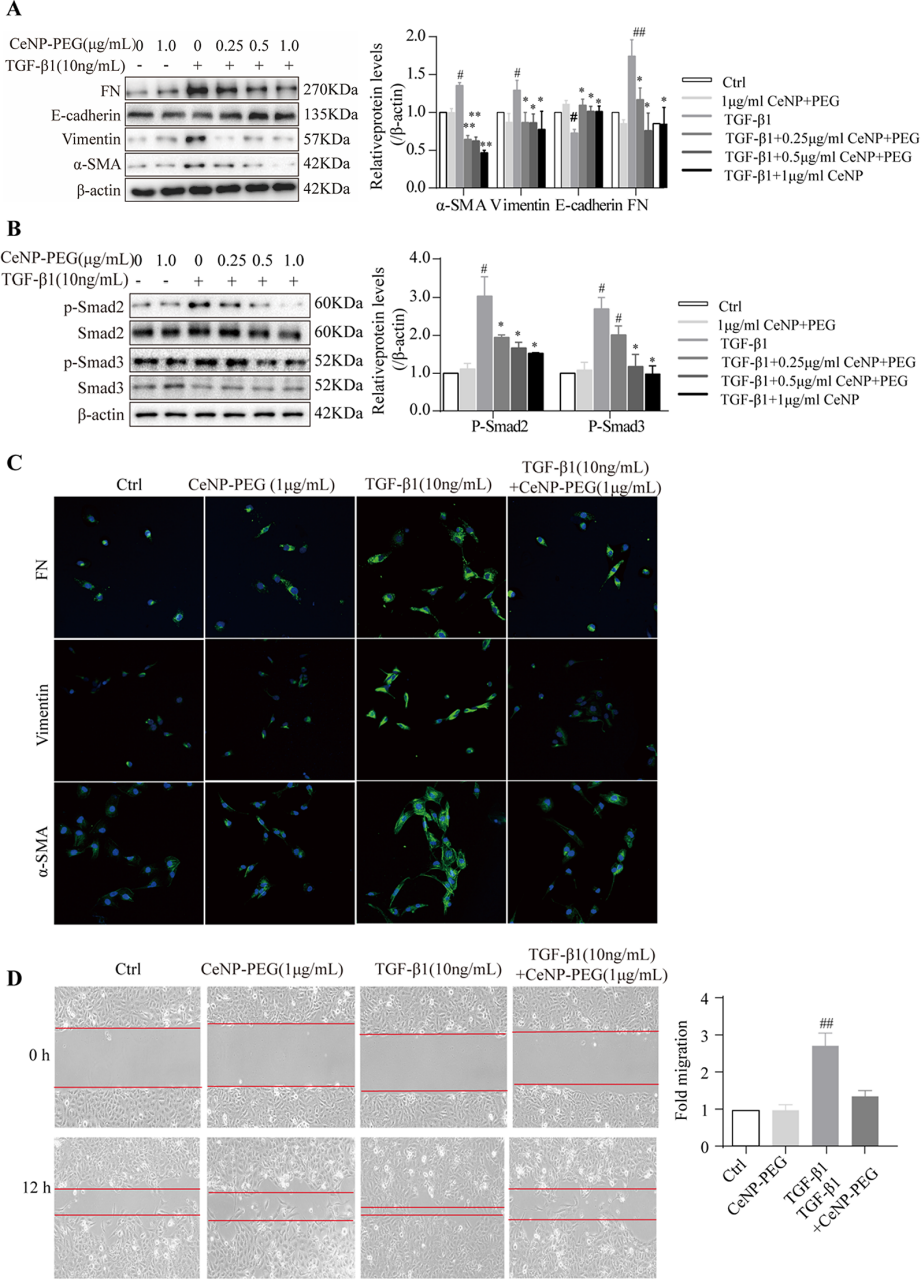
CeNP-PEG protected HK-2 cell from TGF-β1 induced EMT process. **A** The expression of α-SMA, Vimentin, FN and E-cadherin was evaluated by western blot analysis. β-actin was used as the loading control. **B** The expression of phosphorylated and total Smad2 and Smad3 was evaluated by western blot analysis. β-actin was used as the loading control (^#^*P* < 0.05 and ^##^*P* < 0.01 for TGF-β1 *vs.* control, and *P < 0.05 and **P < 0.01 for TGF-β1 *vs.* TGF-β1 + CeNP-PEG; *n* = 3 for each group). **C** The immunofluorescence analysis was performed on α-SMA, Vimentin and FN in HK-2 cells. Original magnification: × 200. All data were presented as mean ± SD. **D** The scratch assays were performed on HK-2 cells treated with TGF-β1 and/or CeNP-PEG, and the wound closure was quantified. The red lines indicate the leading edge after 12 h (*n* = 3 for each group)

Next, the investigators utilized the EMT cell model of TGF-β1-induced HK-2 cells, and evaluated its phenotype alteration and proliferation. In order to investigate the effect of CeNP-PEG on fibrosis in vitro, HK-2 cells were treated with TGF-β1 for 48 h with the presence of CeNP-PEG on different concentrations (0, 0.25, 0.50 and 1.00 μg/mL) or vehicle. Then, the effect of CeNP-PEG on the expression of α-SMA, Vimentin and FN were investigated. The western blot results revealed that the protein levels of α-SMA, Vimentin and FN were markedly upregulated in HK-2 cells after treatment with TGF-β1, when compared to those without TGF-β1 (Fig. 3A). After incubation with TGF-β1 following the addition of 0.5 μg/mL and 1 μg/mL of CeNP-PEG, the α-SMA, Vimentin and FN protein levels significantly decreased, when compared with those untreated with CeNP-PEG (Fig. 3A), while the E-cadherin protein levels significantly increased. The relative densitometric analysis was compared with β-actin, and the result was consistent with that of the immunofluorescence (Fig. 3C). These data show that CeNP-PEG inhibits the TGF-β1-induced phenotypic transition of HK-2 cells from epithelial-to-mesenchymal cells. Previous studies have revealed that the TGF-β1 orchestrating with its downstream Smad signaling pathway plays a crucial role in the development of EMT [22]. Thus, it was determined whether CeNP-PEG could affect the TGF-β1/Smad signaling pathway. The western blot analysis indicated that the p-Samd2/3 protein levels on TGF-β1-induced HK-2 cells were significantly downregulated after the treatment with CeNP-PEG, when compared to those without CeNP-PEG (Fig. 3B). These results suggest that CeNP-PEG significantly blocked the activation of TGF-β1/Smad signaling in vitro. In addition, the cell migration results also revealed that CeNP-PEG can reduce the TGF-β1-stimulated cell migration (Fig. 3D).

### CeNP-PEG blocked the aerobic glycolysis and enhances OXPHOS contributing to EMT suppression in vitro

**Fig. 4:**
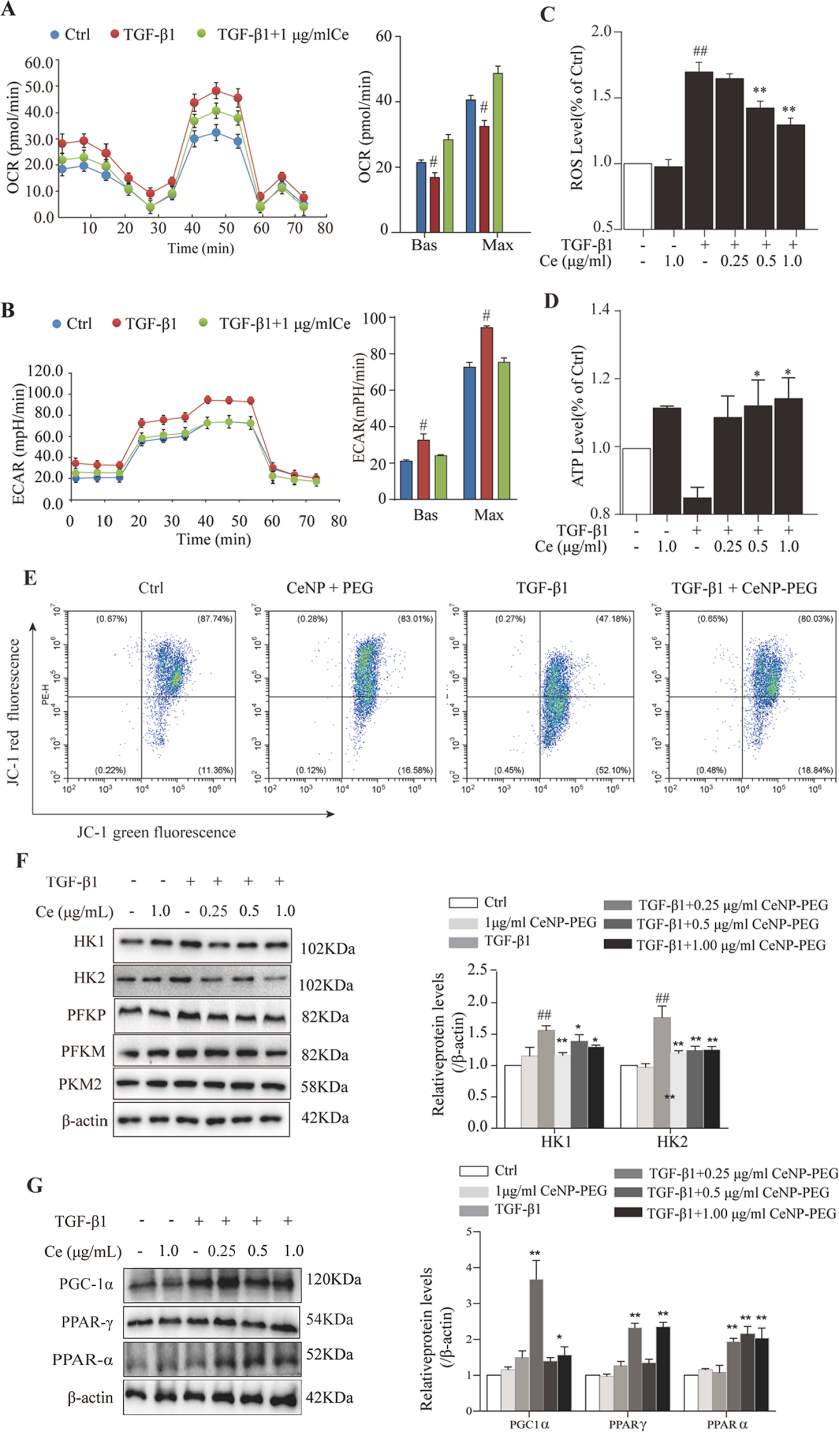
CeNP-PEG blocked the aerobic glycolysis and enhances OXPHOS, contributing to EMT suppression in vitro **A** The OCR measurements of the mitochondrial stress test and **B** The ECAR measurements of the glycolysis stress test were performed in HK-2 cells treated with or without TGF-β1, and/or CeNP-PEG. The basic and maximum capacity of OCR and ECAR were quantified. **C** The ROS levels of cells treated with TGF-β1 and/or CeNP-PEG was determined using the fluorescence spectrophotometry. **D **The ATP content of cells treated with TGF-β1 and/or CeNP-PEG was determined using the ATP Bioluminescence Assay Kit. **E** Mitochondrial membrane potential of cells treated with TGF-β1 and/or CeNP-PEG was measured by flow cytometry. **F** and **G** The expression of HK1/2, PFKP, PFKM and PKM2, total-Samd2/3 and p-Samd2/3 stimulated by TGF-β1 and/or CeNP-PEG was evaluated by western blot analysis. β-actin was used as the loading control, and all data were presented as mean ± SD (^#^*P* < 0.05 and ^##^*P* < 0.01 for TGF-β1 *vs.* control, and ^*^P < 0.05 and ^**^P < 0.01 for TGF-β1 + CeNP-PEG; *n* = 3 for each group)

Previous studies have revealed that CeNP-PEG can scavenge multiple ROS that have mimic the activity of some antioxidant enzymes superioxide dismutase and catalase[20]. In order to investigate the ROS scavenging effects of CeNP-PEG on different concentrations (0, 0.25, 0.50 and 1.00 μg/mL) in HK-2 cells, HK-2 cells were treated with TGF-β1 for 48 h with the presence of CeNP-PEG or vehicle. As shown in Fig. 4C, the results for the extracellular ROS levels were determined by fluorescence spectrophotometry using the oxidant-sensitive dye 2′7-DCF-DA. Higher ROS levels were detected in the supernatant of HK-2 cells after treatment with TGF-β1. However, when these cells were treated with TGF-β1 and received 0.5 μg/mL and 1 μg/mL of CeNP-PEG, the induction of the ROS formation was significantly inhibited. In response to TGF-β1-mediated cell stress, the treatment with CeNP-PEG can maintain the normal levels of mitochondrial ATP (Fig. 4D). The ATP production associated with the loss of mitochondrial antioxidants and peroxisome activity would occur, and result in enhanced ROS generation [23]. These studies suggest that CeNP-PEG may augment the mitochondrial ROS generation. Hence, the cellular respiration base on the ECAR and OCR in HK-2 cells treated with TGF-β1 for 48 h with the presence of CeNP-PEG or vehicle was further examined. The ECAR (Fig. 4B) in HK-2 cells markedly increased, while the OCR (Fig. 4A) markedly decreased after 48 h of treatment with 10 ng/ml of TGF-β1, when compared with those without TGF-β1, and this was in agreement with previous studies [8]. However, the ECAR (Fig. 4B) markedly decreased, while the OCR (Fig. 4A) markedly increased, after treatment with CeNP-PEG, when compared to those untreated with CeNP-PEG. In addition, the JC-1 staining revealed that the mitochondrial membrane potential markedly increased after treatment with CeNP-PEG (Fig. 4E). These shows that CeNP-PEG can block the TGF-β1-induced glycolytic phenotype in HK-2 cells.

Previous studies have revealed that TGF-β1/Smad signaling promotes both fibrotic proteins and glycolytic proteins to increase [24]. In the present study, the correlation between fibrosis and aerobic glycolysis was verified using HK-2 cells treated with or without 10 ng/ml of TGF-β1. Similarly, the western blotting analysis revealed that the expression of glycolytic proteins HK1, HK2, PFKP and PKM2 in HK-2 cells were remarkably upregulated after 48 h of treatment with TGF-β1, when compared with those without TGF-β1(Fig. 4F). However, the expression of HK1 and HK2 was downregulated on TGF-β1-induced HK-2 cells with the presence of CeNP-PEG, when compared with those untreated with CeNP-PEG (Fig. 4F). Next, it was further investigated whether CeNP-PEG could affect the TGF-β1/Smad signaling pathway. Similarly, the results revealed that the p-Samd2/3 protein levels on TGF-β1-induced HK-2 cells were significantly downregulated after the treatment with CeNP-PEG, when compared with those untreated with CeNP-PEG (Fig. 4G). Overall, these results suggest that CeNP-PEG ameliorates the TGF-β1-induced activation, in which OXPHOS switches to the glycolytic program during the process EMT (as summarized in Fig. 6).

### CeNP-PEG blocked the aerobic glycolysis in UUO-induced kidney fibrosis in mice

**Fig. 5:**
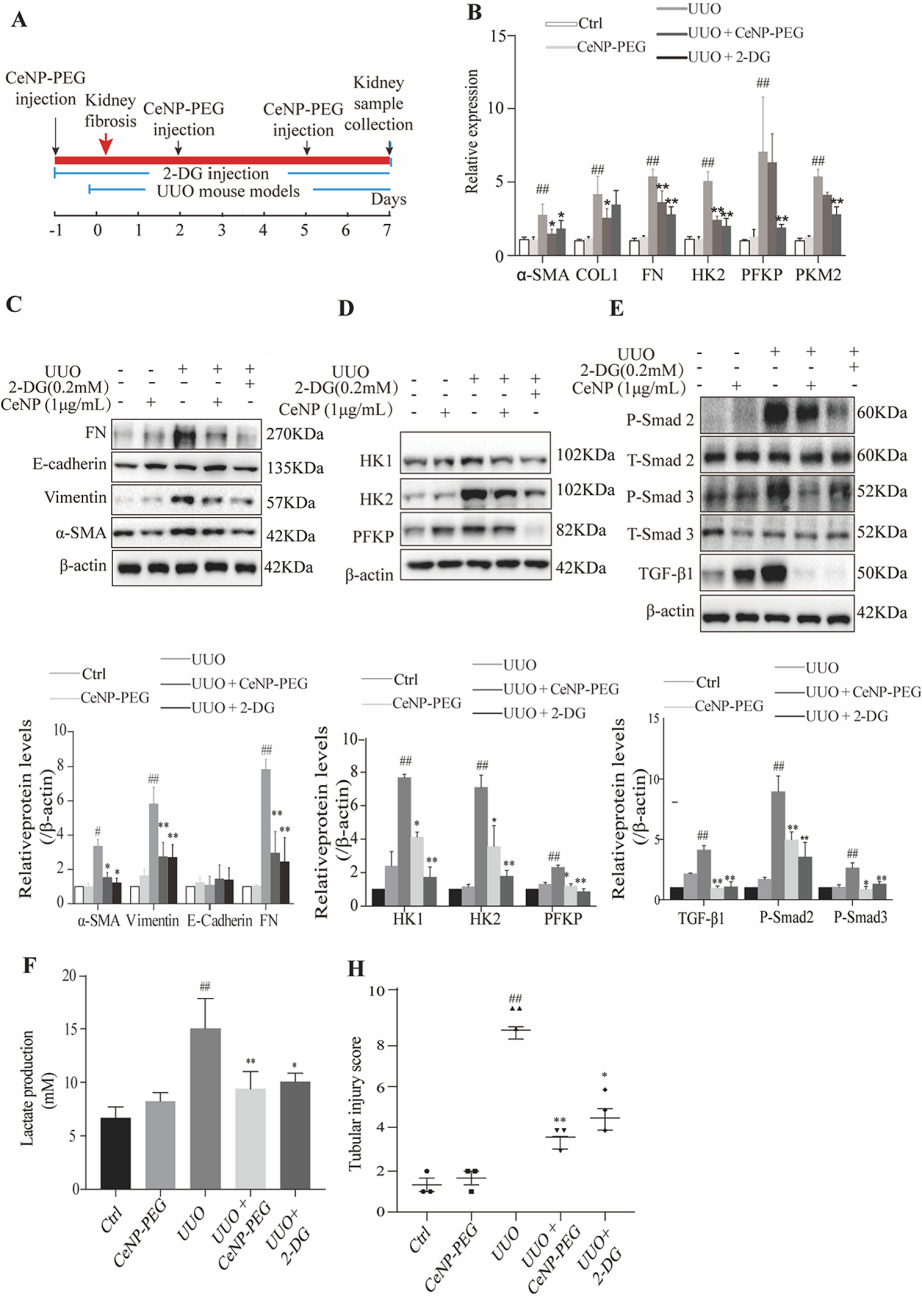

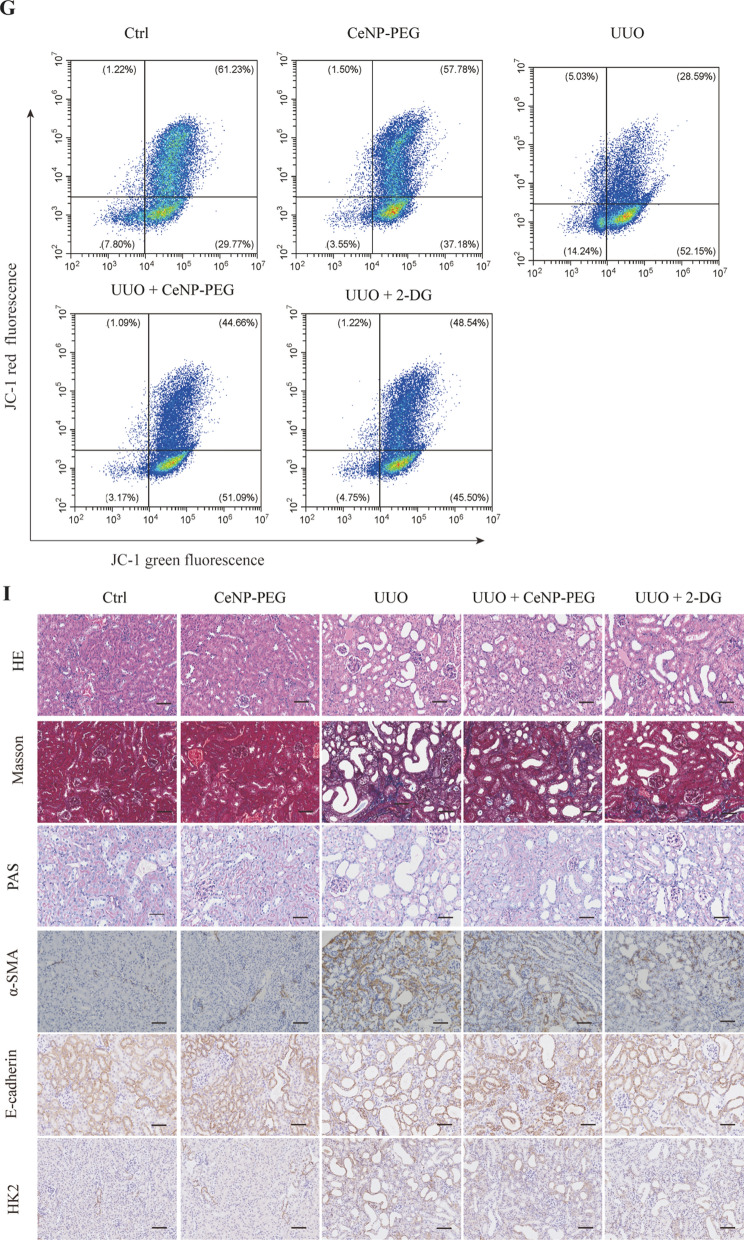
CeNP-PEG inhibited aerobic glycolysis in UUO mice. **A** The schematic graph of the experimental design. **B** The expression of α-SMA, COL1, FN, HK2, PFKP and PKM2 was evaluated by RT-PCR analysis. β-actin was used as the control (^#^*P* < 0.05 and ^##^*P* < 0.01 for UUO *vs.* control, and ^*^P < 0.05 and ^**^P < 0.01 for UUO *vs.* UUO + CeNP-PEG or UUO + 2-DG; *n* = 3 for each group). (**C**, **D** and **E**) The expression of α-SMA, E-cadherin, Vimentin, FN, HK1, HK2, PFKP, and phosphorylated and total Smad2 and Smad3 was evaluated by western blot analysis. β-actin was used as the control. (^#^*P* < 0.05 and ^##^*P* < 0.01 for UUO *vs.* control, and ^*^P < 0.05 and ^**^P < 0.01 for UUO *vs.* UUO + CeNP-PEG or UUO + 2-DG; *n* = 3 for each group). **F** The lactate acid production was measured (^#^*P* < 0.05 and ^##^*P* < 0.01 for UUO *vs.* control, and ^*^P < 0.05 and ^**^P < 0.01 for UUO *vs.* UUO + CeNP-PEG or UUO + 2-DG; *n* = 3 for each group). **G** The mitochondrial membrane potential of cells isolated from UUO kidney in the absence or presence of CeNP-PEG or 2-DG was measured by flow cytometry. **H** The tubular injury scores of UUO mice after different treatments (^#^*P* < 0.05 and ^##^*P* < 0.01 for UUO *vs.* control, and ^*^P < 0.05 and ^**^P < 0.01 for UUO versus UUO + CeNP-PEG or UUO + 2-DG; *n* = 3 for each group). **I** The renal injury was evaluated by H&E staining. The renal fibrosis was evaluated by Masson trichrome and PAS staining, and immunohistochemical analysis was performed to determine the expression of α-SMA, E-cadherin and HK2

As previously reported, the phosphorylation of the main effectors of canonical TGF-β1 signaling, Smad2/3, was also enhanced in UUO kidneys [8]. Thus, it was determined whether the CeNP-PEG treatment blocked the UUO-induced TGF-β1/Smad signaling pathway. The western blotting analysis indicated that the treatment with CeNP-PEG suppressed the p-Smad 2/3 levels, when compared to those without CeNP-PEG (Fig. 5E). Furthermore, the TGF-β1/Smad signaling can promote the metabolic reprogramming. This forces cells to switch from OXPHOS to aerobic glycolysis. Hence, it was determined whether CeNP-PEG could block the TGF-β1-induced glycolytic phenotype in the UUO mice model. Simultaneously, in the present experiment, UUO mice were also treated with 2-DG, and the results were compared with those for UUO mice and sham mice. This was in agreement with previous studies [25]. High levels of glycolytic enzymes mRNA and protein, including HK2, PFKP and PKM2 were found in UUO-induced kidney fibrosis in mice, when compared to the kidney tissues obtained from sham controls (Fig. 5B and D). However, the HK1 and HK2 protein levels significantly decreased after treatment with CeNP-PEG or 2-DG, when compared to untreated UUO mice (Fig. 5B and D). These suggest that CeNP-PEG block the UUO-induced TGF-β1/Smad signaling pathway and aerobic glycolysis activation. The glycolysis related signals by lactate production were further investigated using a lactate assay kit. As shown in Fig. 5F, compared to untreated UUO mice, the lactate production markedly decreased after treatment with CeNP-PEG or 2-DG.In addition, it was determine whether the CeNP-PEG treatment blocked the UUO-induced changes in the mitochondrial membrane potential through the JC-1 dye. Compared to untreated UUO mice, the mitochondrial membrane potential markedly increased after treatment with CeNP-PEG or 2-DG (Fig. 5G). UUO mice presented with a significantly increased Masson staining and PAS staining, while the staining dramatically decreased in the kidneys of CeNP-PEG treated UUO mice animals. As shown in Fig. 5I, the increase in α-SMA and HK2 levels as well as the decrease in E-cadherin levels of fibrotic kidneys was further confirmed by the immunohistochemical staining, while the α-SMA and HK2 expression significantly decreased as well as the E-cadherin expression significantly increased after treatment with CeNP-PEG (Fig. 5I). These suggest that CeNP-PEG blocked the aerobic glycolysis in UUO-induced renal fibrosis in mice.

## Discussion

Metabolic reprogramming is the hallmark of renal fibrosis with multiple dysregulated metabolic pathways, such as aerobic glycolytic, which has been detected in kidney fibrosis [8, 26]. Furthermore, aerobic glycolytic is a critical factor that causes epithelial cell damage and inflammation [27]. Numerous studies have revealed that restoring glycolysis can offer new therapeutic approaches for the prevention and treatment of kidney fibrosis [10, 25, 28]. In addition, metabolic dysregulation is often associated with mitochondrial dysfunction, manifesting the influence of oxidative stress in kidney fibrosis [29, 30]. One of the critical factors is excessive ROS [31]. As a result, scavenging ROS may be warranted for the treatment of renal fibrosis.

**Fig. 6:**
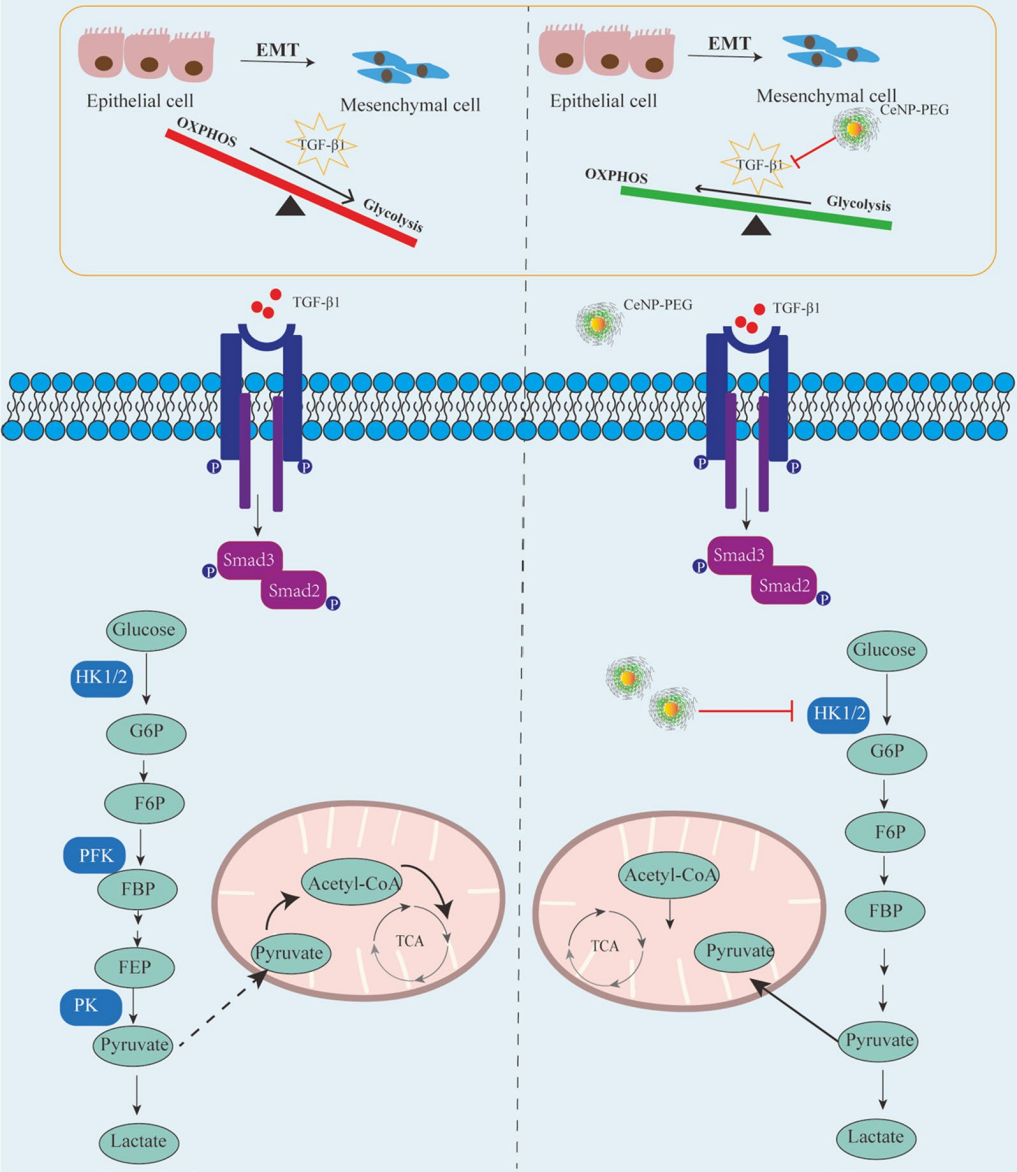
CeNP-PEG reverses the renal fibrosis by blocking the dysregulated metabolic status, in which OXPHOS switches to the aerobic glycolytic program. In normal conditions, renal epithelial cells prefer OXPHOS for energy supply. During the EMT process, TGF-β1 induces the metabolic reprogramming in renal epithelial cells, and takes glycolytic metabolism as the priority to OXPHOS. Furthermore, CeNP-PEG can block this through the suppression of HK1 and HK2 during the EMT process, exerting the protective function in the progress of kidney fibrosis

The present study considered the possibility that CeNP-PEG acts as a potential antioxidant, and protect cells and tissues from several forms of oxidative stress in vitro and in vivo [32, 33]. In the present study, the administration of CeNP-PEG ameliorated the profibrogenic phenotypes induced by TGF-β1 in HK-2 cells in vitro. In addition, the administration of CeNP-PEG also inhibited the ROS-mediated TGF-β1-induced HK-2 cells injury. Furthermore, the increase in mitochondrial ATP generation was observed under CeNP-PEG treatment, in which the ATP production associated with loss of mitochondrial antioxidants and peroxisome activity would occur and result in enhanced ROS generation [34, 35]. TGF-β1 and its downstream Smad signaling pathway plays a crucial role in the development of EMT, and alters the metabolism through mitochondrial injury and aerobic glycolytic [36]. The present study, it was demonstrated that CeNP-PEG increase mitochondrial OXPHOS activity as judged by mitochondrial OCR and also decrease glycolytic activity as judged by ECAR. Since EMT is accompanied by the increased expression of glycolytic enzymes [37, 38].These indicates the inhibition effects of CeNP-PEG on rate-limiting glycolysis enzyme HK1 and HK2 protein levels, which ameliorated the TGF-β1-induced activation, and are associated with the decrease in cellular aerobic glycolysis. These suggests that CeNP-PEG regulate the switch between glycolysis and OXPHOS during the process EMT (as summarized in Fig. 6).

The UUO in the rodents is a classic model of renal interstitial fibrosis, which is similar to patients with renal fibrosis, and has been clinically observed in various aspects [39]. The present study revealed that CeNP-PEG can significantly reduce the α-SMA, Vimentin and FN protein levels, and dramatically decreased the collagen deposition in UUO kidneys. These findings provide the first evidence that CeNP-PEG can ameliorate kidney fibrosis in vivo. In addition, it was verified that CeNP-PEG can suppresses the p-Smad2/3 levels in vivo. These suggest that CeNP-PEG inhibits the EMT and renal fibrosis through Smad-dependent pathways. In the present study, it was demonstrated that the protein levels of HK1 and HK2 significantly decreased after treatment with CeNP-PEG in vivo. Thus, the treatment with CeNP-PEG can decrease the glycolysis-related enzyme expression, and lower the expression of epithelial injury and fibrosis markers. In the present experiment, the treatment for UUO mice with 2-DG, which is a glycolysis inhibitor that effectively mitigates kidney fibrosis, was in accordance with that in previous studies [25]. These suggest that CeNP-PEG can block the UUO-induced TGF-β1/Smad signaling pathway and aerobic glycolysis. This property suggest that the CeNP-PEG inhibition of glycolysis can decrease the extent of renal fibrosis in vivo.

## Conclusions

In summary, these present results indicate that CeNP-PEG suppresses HK1 and HK2 to block the dysregulated metabolic status, in which damaged epithelial cells take glycolytic metabolism as priority to oxidative phosphorylation (OXPHOS**)**, exerting the protective function in the progress of renal fibrosis. To our knowledge, the present study is the first to attempt the application of CeNP-PEG, in order to provide another therapeutic option on renal fibrosis.

## Methods

### Synthesis of ceria nanoparticles

The CeNP were synthesized according to previously reported procedures with slight modifications [16]. Initially, 0.43 g (1.0 mmol) of cerium (III) acetate hydrate and 3.25 g (12 mmol) of oleylamine were dispersed in 15 mL of xylenes, and vigorously stirred at r.t. for two hours. After slowly heating to 90 °C under vacuum, 1 mL of ddH_2_O was swiftly injected into the solution under vigorous stirring, and the mixture color changed to an off-white color, suggesting that a reaction occurred. Then, the mixture was stirred and aged at 90 °C for three hours under Ar protection. Afterwards, the CeNP were precipitated in 100 mL of ethanol after cooling down to room temperature, harvested by centrifugation, and washed for at least five times with ethanol. The purified CeNP were easily dispersible in chloroform for storage and use.

### Synthesis of CeNP-PEG

In order to synthesize the biocompatible CeNP, the CeNP were coated with DSPE-PEG co-polymer via combination of the film hydration method and probe sonication assisted method [20]. Briefly, 50 mg of mPEG^2k^-DPSE (1.85 × 10^–2^ mmol) was dissolved in 2.0 mL of chloroform, and mixed with chloroform containing 10 mg of CeNP. Then, the mixture was stirred for two hours at r.t., and the solvents were completely evaporated using a rotary evaporator at 60 °C under vacuum for 30 min. Next, 10 mL of ddH_2_O was added, and this was dispersed by probe sonication for five minutes at 30% amplitude with full cycle. After filtration to remove the precipitate, the excess mPEG^2k^-DPSE was removed by dialysis against a 10-kDa molecular weight cut-off bag filter. The purified CeNP-PEG was stored in ddH_2_O at 4 °C.

### Characterization

The transmission electron microscope (TEM) images and selected area electron diffraction (SAED) pattern were obtained using the JEOL 2100 (JEOL Ltd., Tokyo, Japan). The TEM was operated at 200 keV. The elemental compositional analysis of CeNPs was carried out using the energy dispersive spectrometer (EDS) system attached to the TEM. The Fourier transform infrared (FT-IR) spectra of the CeNP and CeNP-PEG were analyzed by using an infrared spectrophotometer. The X-ray powder diffraction (XRD) patterns were obtained on a Bruker D8 advance spectrometer (Bruker AXS Inc., Madison, USA). The 2*θ* range was 10–90° with Cu *Kα* radiation (λ = 0.154 nm), and this was operated at 40 mA and 40 kV. Phase identification was performed using the MDI JADE 5.0 software. The X-ray photoelectron spectra (XPS) was recorded using the RBD 147 upgraded PHI 5000C ESCA system (PerkinElmer Inc., MA, USA) equipped with a dual X-ray source, in which the Mg *Kα* (1,253.6 eV) anode and a hemispherical energy analyzer were used. The base pressure during the XPS study was maintained at below 10^–6^ Pa. The measurements were conducted at a pass energy of 93.90 eV, and the binding energy was calibrated using contaminant carbon (C1s = 284.6 eV). The hydrodynamic diameters and zeta potentials of the nanoparticles were measured by dynamic light scattering (DLS) using the Zetasizer Nano-ZS system (Malvern Zetasizer Nanoseries, Malvern, UK), which was performed at 25 °C. The concentration of CeNP-PEG was determined by inductively coupled plasma-atomic emission spectrometry (ICP-AES, Hitachi P-4010, Tokyo, Japan) with a RF power of 1,100 W and a nebulize gas flow of 0.9 L/min. Standard solutions with Ce concentrations of 1, 5, 10, 20, 50, 100 and 200 ppm were prepared, and a calibration curve was made by plotting the corresponding signal peaks *vs.* the Ce concentrations. The CeNP-PEG sample was digested using HNO_3_ (68%) and H_2_O_2_ (30%), until the effervescence stopped, indicating the full dissolution of the ceria. The resulting colorless solution was diluted using double distilled (D.I.) water, and measured by ICP-AES. The concentration of the CeNP-PEG mentioned in the experiments indicate the ceria (Ce) concentration measured by ICP-AES.

### Animals and animal models

Age matched 8–10 week-old male C57BL/6J mice were used throughout the study. The Institutional Animal Care and Use Committee of the University of Fudan approved all the experiments. UUO surgery was performed using an established protocol. Briefly, the ligation of the left ureter under general anesthesia was performed through an abdominal incision, and sham-operated mice were used as normal controls. In the experiments with CeNP-PEG injection, mice were intravenously injected with 0.25 mg/kg, 0.5 mg/kg and 1 mg/kg of CeNP-PEG through the tail vein at one day before the UUO, and at day 2 and 5 after the UUO. The 2-deoxy-D-glucose (2-DG,Cat# D8375; Sigma-Aldrich) was intraperitoneally administered at a dose of 100 mg/kg at one day before the UUO, and at the subsequent seven consecutive days. Then, these mice were euthanized to harvest the blood and kidneys on day seven after the UUO. One portion of the kidney was fixed in 4% paraformaldehyde, followed by paraffin embedding for histological and immunohistochemical staining. Another portion was immediately frozen in liquid nitrogen, and stored at −80 °C for the extraction of RNA and protein.

### Cultured HK-2 cells

Human kidney proximal tubular epithelial (HK-2) cells were obtained from the American Type Culture Collection (ATCC, Rockville, MD, USA). HK-2 cells were maintained in RPMI1640 medium (Thermo Fisher Scientific) with 10% fetal bovine serum (FBS, Gibco), and 100 U/ml of 1% (v/v) penicillin and 100 U/ml of streptomycin at 37 °C with 5% CO_2_ in an incubator. When 80–90% confluence was reached, these cells were trypsinized and subcultured at a 1:3 split ratio in new culture flasks. For the EMT experiment, cells were seeded at a proper density on cell slides in 6-well plates to 40–50% confluence in complete medium, which contained 10% FBS for 12 h. Then, this was changed to a medium that contained 0.5% FBS after washing twice with the medium. After serum starvation for 12–16 h, the CeNP-PEG were added to the 0.5% FBS medium for indicated time periods, and at the indicated concentration at 30 min before the TGF-β1 treatment. Afterwards, these cells were exposed to the treatment for 48 h before harvesting and subjecting these to western blotting and immunofluorescence staining, respectively.

### ROS scavenging activity of CeNP-PEG in vitro

HK-2 cells were treated with100 μM of H_2_O_2_ for 2 h with the presence of the CeNP-PEG at different concentrations (0, 0.25, 0.50 and 1.00 μg/mL) or vehicle. The effect of CeNP-PEG on extracellular ROS scavenging was investigated by DCFH-DA. Briefly, cells were loaded with CM-H2-DCFDA reagent (Invitrogen) by incubating cells with 5 μM of probe solution in PBS for 20 min at 37 °C. Then, the fluorescence microscopy was visualized using a Carl Zeiss LSM710 confocal microscope, and processed using Photoshop software (Adobe Systems, Inc., San Jose, CA, USA).

### Scratch assay

For the scratch assays, 2 × 10^5^ HK-2 cells were seeded in 6-well plates in complete medium. On the next day, the monolayer was disrupted with a sterile 200 ul pipette tip, Then, this was changed to a medium that contained 0.5% FBS after washing twice with the medium. After serum starvation for 12–16 h, 0.5 μg/ml of CeNP-PEG was added to the 0.5% FBS medium at 30 min before the TGF-β1 treatment for 12 h at 37 °C. Then, the cellular leading edge was measured and quantified.

### ROS assay

The extracellular ROS level was determined by fluorescence spectrophotometry using oxidant-sensitive dye 2′7-DCF-DA. The DCFDA assay for intracellular ROS production was performed, as previously described. Briefly, cells were loaded with CM-H2-DCFDA reagent (Invitrogen) by incubating cells with 5 μM of probe solution in PBS for 20 min at 37 °C. Then, the basal fluorescence was read to provide a well-back-ground control, and the test conditions were added to start the assay. The fluorescence intensity was monitored for one hour.

### Adenosine triphosphate (ATP) assay

The cellular ATP content was measured by luminescence using ATP assay (Cat# S0026; Beyotime), according to the product protocol, and normalized for the total protein levels determined by BCA assay. The luminescence was measured by utilizing the Omega Fluostar machine.

### Measurement of the oxygen consumption rate and extracellular acidification rate

The seahorse Bioscience X96 extracellular flux analyzer (Agilent Technologies) was used to measure the rate change of dissolved O_2_ in the medium that immediately surrounded adherent cells cultured in the XF96 V3 cell culture microplate (Seahorse Bioscience). Then, HK-2 cells, which were cultured in RPMI 1640 supplemented with 0.5% FBS, were seeded in the XF96 V3 cell culture microplate at 0.8 × 10^4^ cells per well. Afterwards, these cells were washed and incubated in the base medium (Agilent Technologies) at 37 °C for one hour. The oxygen consumption rates (OCR, pmol.min-1) was measured in real-time using a mitochondria stress test kit, according to manufacturer’s instructions. Sequential compound injections, including oligomycin A (1 μM), FCCP (1 μM) and Rotenone/antimycin A (0.5 μM), were applied on the microplate to test the glycolytic activity. The extracellular acidification rates (ECAR) were measured in real-time using a glycolysis stress test kit, according to manufacturer’s instructions. Sequential compound injections, including glucose (25 mM), oligomycin A (1 μM) and 2-DG (50 mM), were applied on the microplate to test the glycolytic activity.

### Measurement of mitochondrial membrane potential

The mitochondrial membrane potential was determined by JC-1 dye (Cat# C2006; Beyotime), according to the product protocol. Briefly, cultured cells or those separated from the kidney were collected and washed with JC-1 buffer solution for two times. These cells were incubated with JC-1 working stock for 20 min at 37 °C, and washed with JC-1 buffer solution for two times. Then, the cell mitochondrial membrane potential was detected using flow cytometry.

### Biodistribution of CeNP-PEG in tissues of UUO mice

CeNP-PEG were dispersed in saline solution and intravenously administered through the tail vein. The animals were euthanized at 2, 24 and 48 h after the administration of CeNP-PEG, and the major organ was dissected. Then, these organs were diluted with an aqueous solution and analyzed for cerium concentration by ICP-AES to calculate the percentage of injected dose per gram of tissue(% ID/g).

### Lactate production

Lactate production was determined using a lactate assay kit (Cat# KTB 1100; Abbkine). Briefly, the kidney tissue was perfused with cold PBS to remove the red blood cells. The homogenize tissue at 0.10 g/ mL was fixed in cold lactate assay buffer. Afterwards, this was centrifuged at 12,000 g for five minutes at 4 °C. Subsequently, the supernatant was used for the lactate assay.

### Quantitative RT-PCR

The total RNA was extracted from kidney tissues using the Trizol reagent lysis reagent, and precipitated in isopropanol. The complementary DNA (cDNA) was synthesized using primeScript RT Master Mix (Cat# RR036A, Takara, Japan), and TB Green premix Ex Taq was performed using the CFX96 touch Real-Time PCR detection system (Bio-Rad), according to manufacturer’s instructions. The specificity of the RT-PCR was confirmed using the melting-curve analysis. The expression levels of the target genes were normalized by the β-actin level in each sample (*n* = 3). The primers for the qPCR are listed in Additional file 1: Table S1.

### Western blot analysis

After harvesting tissues or cells in the in vivo or in vitro experiments, the kidney tissues were homogenized, and cells were lysed in buffer containing protease and phosphatase inhibitor cocktails. Then, the tissue and cell lysates were centrifuged, and the supernatants were quantified for protein content using BCA. Next, the samples were reduced in SDS sample buffer, and processed for SDS-PAGE analysis, as previously described. Immunoblotting was performed using antibodies against the target α-smooth muscle actin (α-SMA; Cat# ab32575; Abcam, Cambridge, MA, USA), fibronectin (FN; Cat# ab2413; Abcam, Cambridge, MA, USA) and phospho-Smad2 (p-Smad2; Cat #3108; CST),Smad2 (Cat# 3103; CST), phospho-Smad3 (p-Smad3; Cat# 9520; CST),Smad3 (Cat# 9523; CST), E-Cadherin (Cat# ab133597; Abcam, Cambridge, MA, USA) hexokinase 1 (HK1; Cat# ab150423; Abcam, Cambridge, MA, USA) and hexokinase 2 (HK2; Cat# ab131196; Abcam, Cambridge, MA, USA), PFKP (Cat# ab204131; (Abcam, Cambridge, MA, USA), PFKM (Cat# ab154804; Abcam, Cambridge, MA, USA), and PKM2 (Cat# 4053; CST), PPARα (Cat# ab245119; Abcam), PPARγ (Cat# ab178860; Abcam), PGC1α(AF7736;Beyotime), and β-actin (Cat# 20,536–1-AP; Proteintech). Horseradish peroxidase (HRP)-tagged secondary antibodies were used for the primary antibody target detection. The ECL substrate (Thermo Fisher Scientific) was used to visualize the protein bands. Densitometry analysis was performed using the Un-Scan-It gel analysis software (Silk Scientific, Orem, UT,USA), as previously described.

### Immunofluorescence staining

HK-2 cells cultured on cover slips were fixed in 4% formaldehyde for 15 min, permeabilized in 0.25% Triton X-100 for 10 min, and blocked in quick blocking liquid for one hour at room temperature. Then, the primary antibody for α-SMA (1:200) or Vimentin or FN (1:100) was incubated overnight at 4 °C. Next, cells were rinsed for three times with PBS containing 0.02% Tween 20. Then, the secondary Alexa Fluor 488 goat anti-rabbit IgG (CST, USA) was incubated at a dilution of 1:200 for one hour at room temperature. After rinsing for three times with PBS containing 0.02% Tween 20, these cells were incubated with DAPI (2 mg/ml) for 1 min. Then, these cells were rinsed for three times with PBS containing 0.02% Tween 20, and cells on the cover glass were mounted on microscope slides with the Prolong Gold anti-fade reagent (Invitrogen, USA). Images were taken using a Carl Zeiss LSM710 confocal microscope, and processed using the Photoshop software (Adobe Systems Inc., San Jose, CA, USA).

### Tissue section analysis

The kidneys were fixed in 4% paraformaldehyde and embed in paraffin. Then, the fixed tissues were embedded in paraffin and sectioned into 4-μm thick slices for hematoxylin and eosin (H&E), Masson trichrome, and periodic acid schiff (PAS). Afterwards, the H&E-stained slides were subjected to histologic evaluation, the × 20 magnification of the images of the renal cortex, and evaluation. The semi-quantitative scoring (0–4) for tubular injury was performed in terms of tubular dilation, proteinaceous cast formation, and loss of brush border. In order to quantify the Masson trichrome-stained collagen-rich area, the tiling imaging system was used.

### Immunohistochemistry (IHC)

The kidney specimens were fixed in 4% paraformaldehyde and embed in paraffin. Then, the fixed tissues were embedded in paraffin, and sectioned into 4-μm-thick slices for IHC staining. After deparaffinization and rehydration, these sections were incubated for 15 min at 95 °C in 0.01 M of citrate buffer (pH 6.0) for antigen retrieval. After incubation with 3% H_2_O_2_, followed by 5% bovine serum albumin (BSA) to avoid nonspecific immunoreactions, the sections were stained with anti-α-SMA, E-cadherin and the HK2 antibody overnight at 4 °C (1:100 dilutions, Abcam). Then, these sections were washed with 0.01 M of PBS, and subsequently incubated using the corresponding secondary antibody (1:200 dilution;8125, CST) for one hour at room temperature. The immunoreactivity was visualized by treatment using the Dako Envision kit HRP (K4006, Dako). Finally, counterstaining was carried out with hematoxylin staining, dehydrating and mounting. The α-SMA-positive cells were observed using the 3,3′-diaminobenzidine-enhanced liquid substrate system, and quantified using a bright microscope (Olympus, CX21FS1, Japan).

### Systemic toxicity assessment in vivo

The systemic toxicity of CeNP-PEG was assessed after treatment with CeNP-PEG (10 mg/kg). The major organs and blood samples were harvested after 21 days post-injection.

### Statistical analyses

All data were presented as mean ± standard deviation (SD). T-test was used to analyze the differences between two groups, and ANOVA was used to analyze the intergroup differences. A *P*-value of < 0.05 were considered statistically significant. The analysis was performed using GraphPad Prism 5 (GraphPad software).

## Supplementary Information

**Additional file 1.** Additional figures and table.

## Data Availability

All data generated or analyzed during this study is available from the corresponding author on reasonable request.

## Abbreviations

2-DG: 2-Deoxy-D-glucose
ATP: Adenosine triphosphate
CeNP: Ceria nanoparticles
CKD: Chronic kidney disease
DLS: Dynamic light scattering
ECAR: Extracellular acidification rates
EDS: Energy dispersive spectrometer
EMT: Epithelial-to-mesenchymal transition
FAO: Fatty acid oxidation
FN: Fibronectin
FT-IR: Fourier transform infrared
H&E: Hematoxylin and eosin
HK1: Hexokinase 1
HK2: Hexokinase 2
ICP-AES: Inductively coupled plasma-atomic emission spectrometry
OCR: Oxygen consumption rates
OXPHOS: Oxidative phosphorylation
ROS: Reactive oxygen species
SAED: Selected area electron diffraction
TEM: Transmission electron microscope
TGF-β1: Transforming growth factor beta1
UUO: Unilateral ureter obstruction
XPS: X-ray photoelectron spectra
XRD: X-ray powder diffraction
α-SMA: α-Smooth muscle actin
