# Correction: Ceria nanoparticles ameliorate renal fibrosis by modulating the balance between oxidative phosphorylation and aerobic glycolysis

**DOI:** 10.1186/s12951-024-03089-w

**Published:** 2025-01-28

**Authors:** Mengling Wang, Feng Zeng, Fengling Ning, Yinhang Wang, Shilin Zhou, Jiaqi He, Cong Li, Cong Wang, Xiaolin Sun, Dongliang Zhang, Jisheng Xiao, Ping Hu, Svetlana Reilly, Hong Xin, Xudong Xu, Xuemei Zhang

**Affiliations:** 1 Department of Pharmacology, School of Pharmacy, Minhang Hospital, Fudan University, Shanghai, 201203 China; 2 Artemisinin Research Center, Institute of Science and Technology, The First Clinical Medical School, Lingnan Medical Research Center, The First Affiliated Hospital, Guangzhou University of Chinese Medicine, Guangzhou, 510405 China; 3 Key Laboratory of Smart Drug Delivery, Ministry of Education, School of Pharmacy, Fudan University, Shanghai, China, Academy for Engineering and Technology, Fudan University, 20 Handan Road, Yangpu District, Shanghai, 200433 China; 4 Science and Technology Innovation Center, Guangzhou University of Chinese Medicine, Guangzhou, 510405 China; 5 Division of Cardiovascular Medicine, Department of Medicine, University of Oxford, John Radcliffe Hospital, Radcliffe, Oxford UK

**Correction: Journal of Nanobiotechnology (2022) 20:3** 10.1186/s12951-021-01122-w

 The authors’ have mistakenly used β-actin gel images in Fig. 3A, Fig. 4G and Fig. 5C. To rectify this error and ensure the integrity of the study, the authors provided the correct figures.
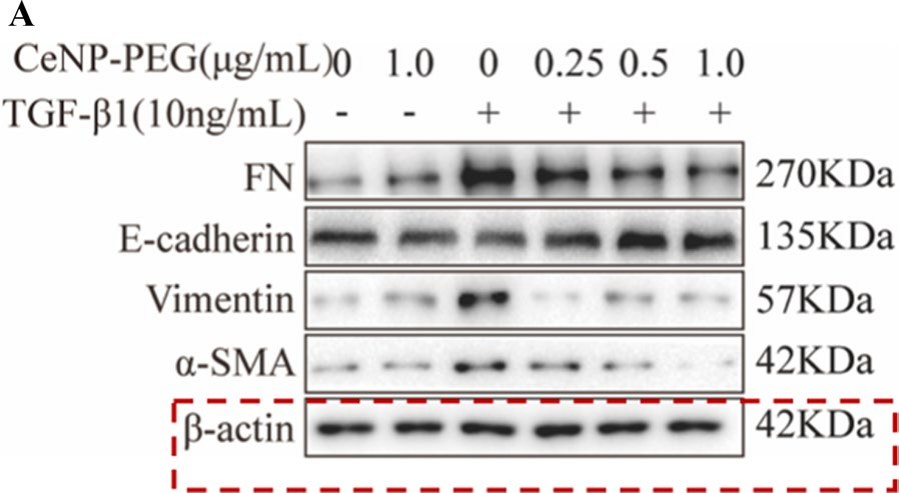


Incorrect Fig. 3A
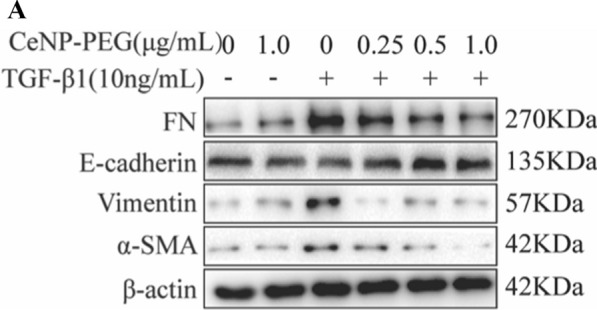


Correct Fig. 3A

**Fig. 3** CeNP-PEG protected HK-2 cell from TGF-β1 induced EMT process. A The expression of α-SMA, Vimentin, FN and E-cadherin was evaluated by western blot analysis. β-actin was used as the loading control. B The expression of phosphorylated and total Smad2 and Smad3 was evaluated by western blot analysis. β-actin was used as the loading control (^#^P < 0.05 and ^##^P < 0.01 for TGF-β1 vs. control, and ^*^P < 0.05 and ^**^P < 0.01 for TGF-β1 vs. TGF-β1 + CeNP-PEG; n = 3 for each group). C The immunofuorescence analysis was performed on α-SMA, Vimentin and FN in HK-2 cells. Original magnifcation: ×200. All data were presented as mean ± SD. D The scratch assays were performed on HK-2 cells treated with TGF-β1 and/or CeNP-PEG, and the wound closure was quantifed. The red lines indicate the leading edge after 12 h (n = 3 for each group)
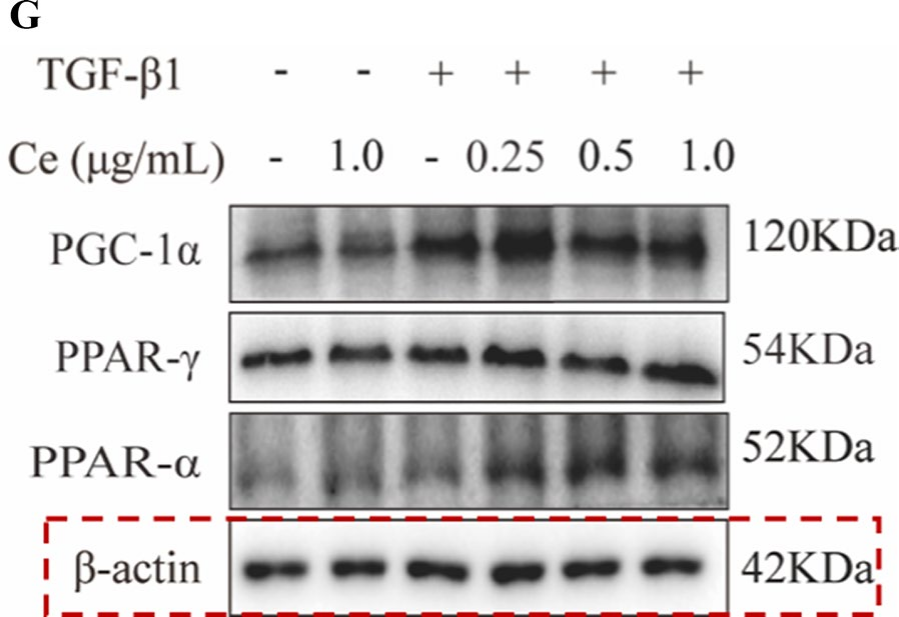


Incorrect Fig. 4G
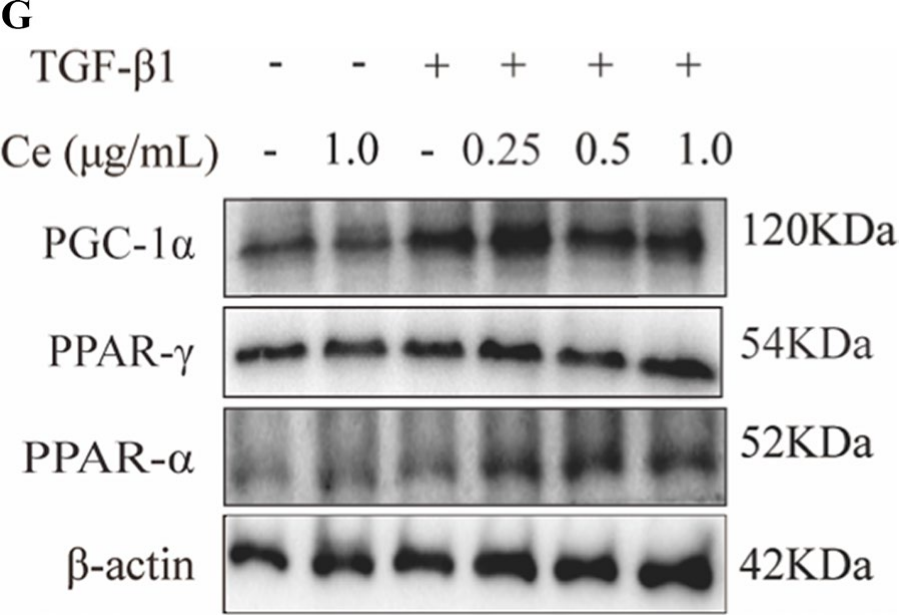


Correct Fig. 4G

**Fig. 4** CeNP-PEG blocked the aerobic glycolysis and enhances OXPHOS, contributing to EMT suppression in vitro A The OCR measurements of the mitochondrial stress test and B The ECAR measurements of the glycolysis stress test were performed in HK-2 cells treated with or without TGF-β1, and/or CeNP-PEG. The basic and maximum capacity of OCR and ECAR were quantified. C The ROS levels of cells treated with TGF-β1 and/or CeNP-PEG was determined using the fluorescence spectrophotometry. D The ATP content of cells treated with TGF-β1 and/or CeNP-PEG was determined using the ATP Bioluminescence Assay Kit. E Mitochondrial membrane potential of cells treated with TGF-β1 and/or CeNP-PEG was measured by flow cytometry. F and G The expression of HK1/2, PFKP, PFKM and PKM2, total-Samd2/3 and p-Samd2/3 stimulated by TGF-β1 and/or CeNP-PEG was evaluated by western blot analysis. β-actin was used as the loading control, and all data were presented as mean ± SD (^#^*P* < 0.05 and ^##^*P* < 0.01 for TGF-β1 vs. control, and ^*^P < 0.05 and ^**^P < 0.01 for TGF-β1 + CeNP-PEG; *n* = 3 for each group)
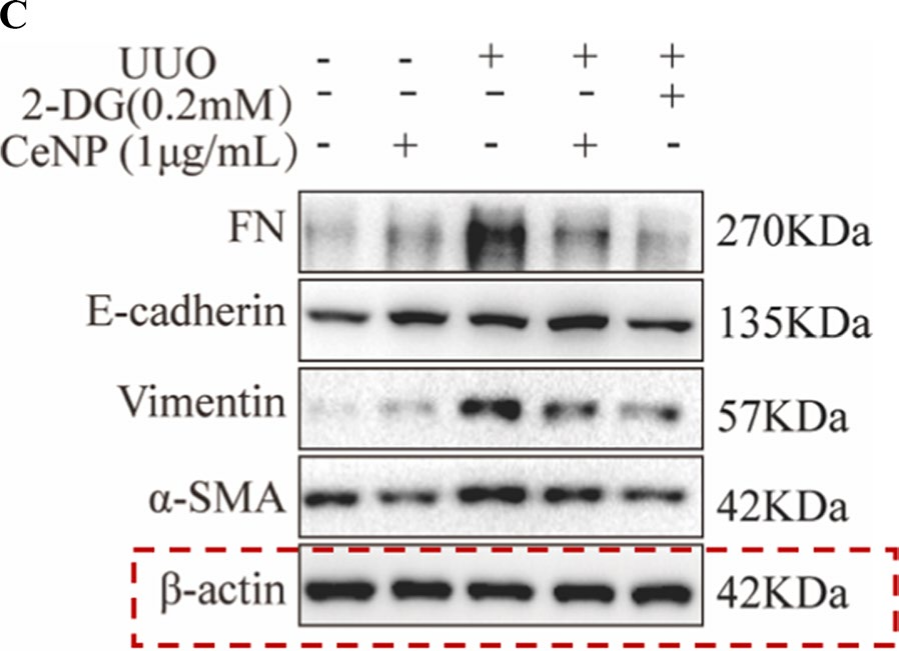


Incorrect Fig. 5C
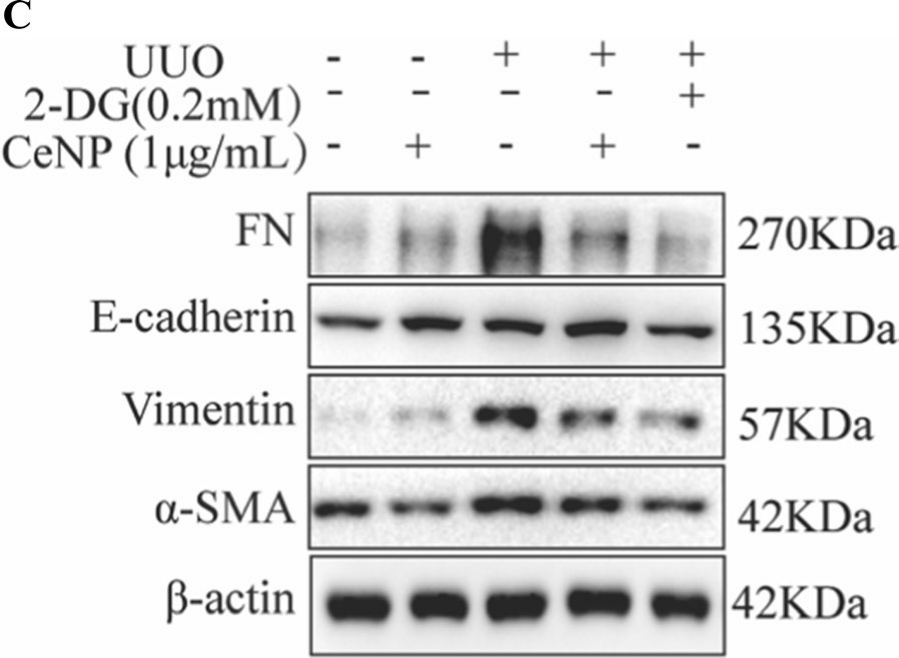


Correct Fig. 5C

**Fig. 5** CeNP-PEG inhibited aerobic glycolysis in UUO mice. A The schematic graph of the experimental design. B The expression of α-SMA, COL1, FN, HK2, PFKP and PKM2 was evaluated by RT-PCR analysis. β-actin was used as the control (^#^*P* < 0.05 and ^##^*P* < 0.01 for UUO vs. control, and ^*^P < 0.05 and ^**^P < 0.01 for UUO vs. UUO + CeNP-PEG or UUO + 2-DG; *n* = 3 for each group). (C, D and E) The expression of α-SMA, E-cadherin, Vimentin, FN, HK1, HK2, PFKP, and phosphorylated and total Smad2 and Smad3 was evaluated by western blot analysis. β-actin was used as the control. (^#^*P* < 0.05 and ^##^*P* < 0.01 for UUO vs. control, and ^*^P < 0.05 and ^**^P < 0.01 for UUO vs. UUO + CeNP-PEG or UUO + 2-DG; *n* = 3 for each group). F The lactate acid production was measured (^#^*P* < 0.05 and ^##^*P* < 0.01 for UUO vs. control, and ^*^P < 0.05 and ^**^P < 0.01 for UUO vs. UUO + CeNP-PEG or UUO + 2-DG; *n* = 3 for each group). G The mitochondrial membrane potential of cells isolated from UUO kidney in the absence or presence of CeNP-PEG or 2-DG was measured by flow cytometry. H The tubular injury scores of UUO mice after different treatments (^#^*P* < 0.05 and ^##^*P* < 0.01 for UUO vs. control, and ^*^P < 0.05 and ^**^P < 0.01 for UUO versus UUO + CeNP-PEG or UUO + 2-DG; *n* = 3 for each group). I The renal injury was evaluated by H&E staining. The renal fibrosis was evaluated by Masson trichrome and PAS staining, and immunohistochemical analysis was performed to determine the expression of α-SMA, E-cadherin and HK2

The correct figures and captions have been included in this correction, and the original article has been corrected.

