# Supplementary material for: Ceria nanoparticles ameliorate renal fibrosis by modulating the balance between oxidative phosphorylation and aerobic glycolysis
Source: J Nanobiotechnology. 2022 Jan 4;20:3. doi: 10.1186/s12951-021-01122-w (PMC8725394; doi:10.1186/s12951-021-01122-w)
Supplement: Supplementary file 1 — Additional file 1. Additional figures and table. [file 12951_2021_1122_MOESM1_ESM.pdf]

Ceria nanoparticles ameliorate renal fibrosis by modulating the balance between oxidative phosphorylation and aerobic glycolysis

Mengling Wang *et al.*

Additional figures

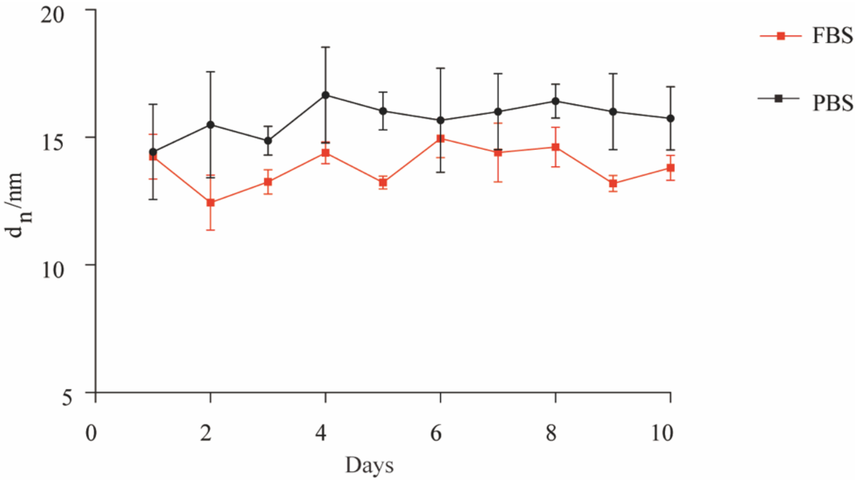

Figure S1. Measurement of the colloidal stability of CeNP-PEG. The hydrodynamic diameters of CeNP-PEG in RPMI1640 medium containing 10% FBS or PBS at different time points (n = 3 independent experiments).

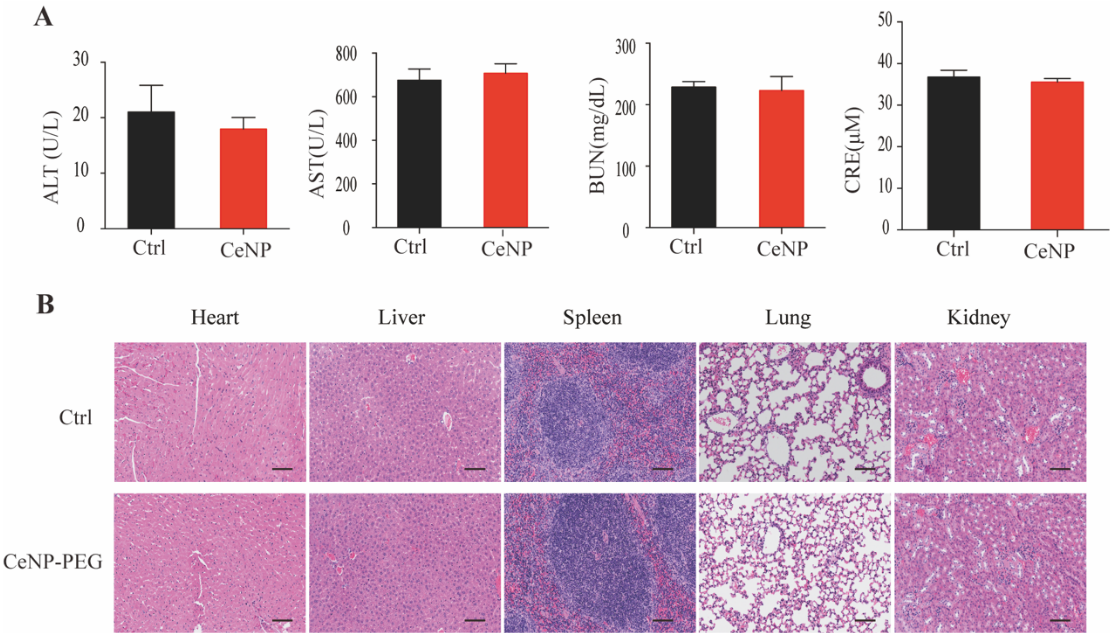

Figure S2. The CeNP-PEG had negligible toxicity in primary organs. (A)The blood analysis of BUN, CRE, ALT and AST. (B) The H&E sections of heart, liver, spleen, lung and kidney after treatment with CeNP-PEG (10 mg/kg) or saline for 21 days. n = 3 independent animals.

Table S1. RT-qPCR primer sequences.

genes	F	R	Species
α-SMA	TGCTGACAGAGGCACCACTGAA	CAGTTGTACGTCCAGAAGGC	Mouse
FN	ACTGTACTTCTGAGGGTCGC	ACTTGGTGATGTGTGAAGGC	Mouse
COL1	CCTCGGGTATTGCTGGACAAC	CAGAAGGACCTTGTTTGCCAGG	Mouse
HK2	TACCACACACCCTACAGCAG	CTCGGGAATGGCGTAGATCT	Mouse
PFKB	GTTCAGAGTGGAGCGACTTG	TGCCACAGAAGTCATTGTCG	Mouse
PKM2	GCTCTAGGTATCGCAGCAGG	GTAAGCGTTGTCCAGGGTGA	Mouse
β-actin	CATTGCGACAGGATGCAGAAGG	TGCTGGAAGGTGGACAGTGAGG	Mouse
